# Are we ready to face the next wave of RSV surge after the COVID-19 Omicron pandemic in China?

**DOI:** 10.3389/fcimb.2023.1216536

**Published:** 2023-12-12

**Authors:** Wujun Jiang, Sainan Chen, Meng Lv, Zhen Zhang, Zhihui Wang, Xuejun Shao, Shenghao Hua, Chuangli Hao, Yuqing Wang

**Affiliations:** ^1^ Department of Respiratory Medicine, Children’s Hospital of Soochow University, Suzhou, China; ^2^ Clinical Laboratory, Children’s Hospital of Soochow University, Suzhou, China

**Keywords:** respiratory syncytial virus infection, COVID-19 pandemic, children, surveillance, intensive care unit

## Abstract

**Background:**

China had its first wave of COVID-19 in 2020 and second wave of COVID-19 Omicron in 2022. The number of RSV cases decreased sharply in 2020 and 2022. Investigation of the resurge of RSV infections after the first wave of COVID-19 will guide us to take preventive actions before the resurge of RSV infections after the second wave of COVID-19 Omicron.

**Methods:**

We analysed epidemiological and clinical data of 59934 patients with lower respiratory tract infections (LRTI) from a prospective long-term cohort surveillance programme in Suzhou, China, collected from February 2016 to January 2022. The annual incidence of RSV infection in children aged<16 years in 2020 and 2021 was compared with the pre-pandemic years 2016 to 2019. We also compared the clinical characteristics, and RSV-related ICU admissions between pre-pandemic years and 2021.

**Results:**

Among children with LRTI, the positive rate of RSV increased by 70.7% in 2021 compared to the average level in the pre-pandemic years. The RSV resurge in 2021 was most prominently in children aged 2-4 years (a significant rise compared with the expected value 149.1%; 95%CI, 67.7% to 378%, P<.01). The percentage of RSV-related ICU admissions decreased in 2021 (3.2% vs 6.7%, P<0.01). The death rate of RSV infections in 2021 was 0.2%, while that in pre-pandemic years was only 0.02%. RSV-associated death in immunocompetent children (complicated by necrotizing encephalitis) was firstly occurred in 2021.

**Conclusions:**

Our findings raise concerns for RSV control in Southeast China after the COVID-19 pandemic especially for children aged 2-4 years. Although ICU admissions were significantly reduced in this resurgence, we could not ignore the increase of RSV-associated death.

## Introduction

Coronavirus disease (COVID-19) has had a substantial impact on the epidemiology and characteristics of respiratory syncytial virus (RSV) infection during 2020 and 2021 in China and worldwide (Edwards, 2021; Tang et al., 2021; Yeoh et al., 2021; Saravanos et al., 2022). RSV is the most common pathogen responsible for low respiratory tract infections (LRTIs) among children aged<5 years, with typical annual seasonality (Shi et al., 2017).

Previous reports have revealed reduction in the incidence of RSV infection of up to 98% in 2020 following the onset of the COVID-19 pandemic (Britton et al., 2020). However, studies from Australia and the U.S. have reported that in 2021, the number of cases of RSV infection began to increase during the spring months as physical distancing restrictions were gradually relaxed, and peaked in the summer, instead of the typical seasonal pattern of peaking during the autumn and winter months. These studies also found that children with RSV infections tended to be older and less likely to have severe disease (Agha and Avner, 2021; Foley et al., 2021). However, these initial studies were performed in 2020-2021 RSV season. It was unclear whether the 2021–2022 RSV season would continue to be increased or just delayed.

China had its first wave of COVID-19 in Jan 2020. The number of RSV cases decreased sharply in 2020, while increased substantially in 2021. Now China is experiencing a second large wave of COVID-19 Omicron starting from Jan 2022. RSV cases decreased again after intense nonpharmaceutical interventions were performed. Investigation of the resurge of RSV infections after the first wave of COVID-19 will guide us to take preventive actions before the resurge of RSV infections after the second wave of COVID-19.

The detection rate of RSV in Beijing, China from February to May 2020 (after COVID-19 pandemic) decreased by 92.17% compared with the same period in previous years (Zhang et al., 2021). However, with the downgrade of prevention level and the opening of schools, it is reported that the positive rate of RSV has been increasing month by month since June in Changsha, China (Lili et al., 2021). By August 2020, the detection rate of RSV reached 12.51%, which is more than six times that of 2019 (Lili et al., 2021). There are only observational studies on the epidemiology of RSV in China, but the clinical features of the newly resurge of RSV infections and its potential threat to children have never been thoroughly investigated after COVID-19 pandemic.

Here, we combined clinical data from two branches of a tertiary paediatric hospital to describe the change in the pattern of RSV in 2020 and 2021 and compare the epidemiology and clinical characteristics of RSV infections to those in the 4 years preceding the onset of the COVID-19 pandemic (2016–2019). The aim of this study is to report and discuss the impact of the COVID-19 pandemic on the change of RSV among hospitalized children with LRTI in Suzhou, China, and to determine if the shifted RSV epidemic was larger or more severe compared with previous years or if age-specific changes were associated with the resurgence.

## Patients and methods

### Study design and setting

The study population comprised all children aged<16 years hospitalised with LRTI in two branches (General Hospital and Jingde Road Branch) of the Children’s Hospital of Soochow University from February 2016 to January 2022. The Children’s Hospital of Soochow University is the only tertiary children’s hospital in Suzhou, southeast China, and it serves the majority of children living in this region. The data from our study were obtained from a prospective long-term cohort surveillance programme.

### COVID-19 and public health measures in Suzhou, China

The COVID-19 pandemic is a public health emergency, given its rapid spread and high incidence. The first COVID-19 case in China was reported in December 2019. Suzhou is a city located in the southeast of China. In Suzhou, the first case was reported in January 2020. On 24 January 2020, an official national lockdown was implemented in China and a level 1 public health emergency response was initiated, which meant enforcing the most intense nonpharmaceutical interventions including a stay-at-home order, prohibition of gatherings, and closure of nonessential businesses, schools, restaurants and hotels. On 24 February 2020, Suzhou downgraded the public health response to level 2, which meant that human movement was allowed in areas with no cases, and restaurants were open with limited capacity. Schools, cinema, and sports halls remained closed. On 27 March 2020, Suzhou downgraded the public health response to level 3. Businesses and recreational activities resumed, and schools were partially reopened. On 1 September 2020, the schools were fully reopened. In Suzhou, only 87 COVID-19 cases were detected in January and February 2020, and no cases were reported from March 2020 to January 2022 (**Supplementary Figure 1**).

### Clinical specimens

Nasopharyngeal aspirates were obtained from all inpatients within 24 hours of admission. Total nucleic acid (DNA and RNA) was extracted from the collected specimens. The nucleic acid extracts were tested for six viruses, including RSV, human rhinovirus (HRV), influenza virus (IFV), parainfluenza virus (PIV), adenovirus (ADV), and human metapneumovirus (HMPV), using multiple respiratory pathogen panel assays (Health Gene Technologies, Ningbo, China). Details regarding the testing for multiple respiratory viruses are given in the **Supplementary Materials**.

### Age groups

Age was categorized and analysed in five age groups as follows: 0-5 months, 6-11 months, 12-23 months, 2-4 years and 5-15 years.

#### Controls

We set an internal control to exclude the possible change in the populations in our area over the years. Eligible controls were those who had hernia and hospitalized in our general surgery department from February 2016 to January 2022. Controls were excluded if they had respiratory symptoms within 14 days before enrollment.

### Definitions

“Severe infections at admissions” was defined as infections requiring supplemental oxygen or mechanical ventilation. If the patient received more than one type of oxygen support, only the higher level of oxygen support was counted (i.e. invasive mechanical ventilation (IMV)>high flow nasal cannula>other oxygen support). Comorbidities, consisting of 9 categories (cardiovascular, respiratory, neuromuscular, renal, gastrointestinal, hematologic or immunologic, metabolic, other congenital or genetic and malignancy), were defined by the pediatric complex chronic conditions classification system version 2 (Feudtner et al., 2014). To better express the change in the pattern of RSV infection since the start of the pandemic, two periods in 2020/2021 were defined according to the timeline of major public health events (national lockdown and school fully reopening) during the COVID-19 epidemic and the seasonal pattern of RSV infection: Phase I (1 February to 31 August) and Phase II (1 September to 31 January of the following year, **Supplementary Figure 2**). Phases I and II were also defined based on the corresponding months in 2016–2019, starting from 1 February 2016 and ending on 31 January 2020.

### Statistical analysis

The proportion of tests positive for RSV during the pandemic years, 2020 and 2021, were compared with the average level during the pre-pandemic years for Phase I and II by calculating the percentage change as:


$$\left[P_{pan}\left(t\right)\;-\;P_{pre}\left(t\right)\right]\;/\;P_{pre}\left(t\right)\times \;100\%$$

where *P_pan_(t)* represents the positive rate in phase *t* of the pandemic year 2020 (February 2020 to January 2021) or 2021 (February 2021 to January 2022), and *P_pre_(t)* represents the average positive rate in phase *t* of the pre-pandemic years (February 2016 to January 2020).

Clinical characteristics were compared between the pandemic 2021 and the pre-pandemic years, 2016–2019 using the Pearson χ^2^ (Yeoh et al., 2021) test or the Mann-Whitney U test, as appropriate. Multivariable logistic regression was performed to calculate the adjusted ORs and 95% CIs. We did not adjust for covariates because our study was to compare the temporal trends rather than the causal relationship between calendar years and outcomes (Cuzick, 1985). We performed a time series analysis to forecast RSV-related admissions counts and their 95% CIs in 2020 and 2021 based on the annual counts from 2016 to 2019. Linear models were used depending on the pattern of change in the counts before 2020 and model fit indices. We compared the observed and forecasted counts in 2020 and 2021 by calculating the percent difference between these 2 numbers using the following equation:


(observed frequency-expected frequency)/expected frequency×100%

All analysis was performed using SPSS Statistics (version 22.0, IBM Corp., Armonk, NY, USA). Statistical significance was set at *P*<0.05.

### Ethics

The study was approved by the Medical Ethics Committee of the Children’s Hospital of Soochow University (2013002).

## Results

**Figure 1:**
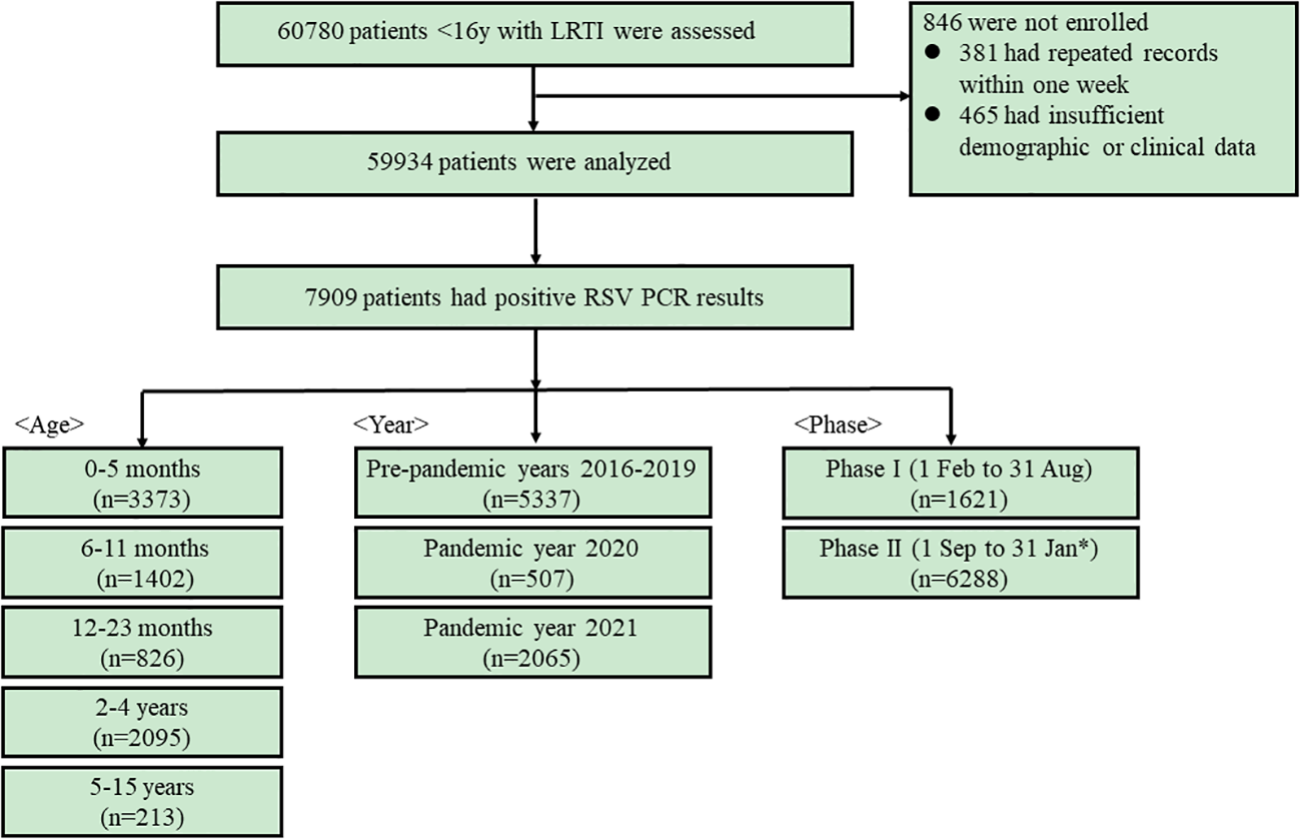
The flowchart of the number of cases included in the study.

**Figure 2:**
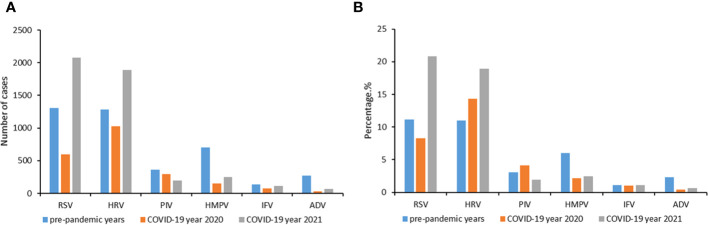
Change of cases number **(A)** and positive rates **(B)** during the COVID-19 pandemic year 2020 and 2021 compared to the average incidences during pre-pandemic years 2016−2019. A study year is defined as February 1st of the current year to January 31st of the following year instead of the traditional calendar year. Six respiratory viruses were tested: respiratory syncytial virus (RSV), human rhinovirus (HRV), influenza virus (IFV), parainfluenza virus (PIV), adenovirus (ADV) and human metapneumovirus (HMPV).

From 1 February 2016 to 31 January 2022, the epidemiological and clinical data were collected on 59934 patients with LRTIs (**Figure 1**). The number of patients tested in pre-pandemic years 2016−2019, pandemic year 2020 and 2021 were 43268, 6833 and 9833, respectively (**Supplementary Figure 3**). The baseline characteristics of these patients from 2016 to 2021 are listed in **Supplementary Table 1**. In 2020, there were significant reductions in the number of cases from the pre-pandemic years for all the viruses tested, while in 2021, a large increase in the number of RSV and HRV cases was observed (**Figure 2**).

Of the 59934 patients with LRTIs, 7909 (13.2%) patients had RSV infections (**Figure 1**). The number of RSV patients in pre-pandemic years 2016-2019, pandemic year 2020 and 2021 were 1005 (12.7%),1287 (16.3%), 1620 (20.5%), 1425 (18.0%), 507 (6.4%) and 2065 (26.1%), respectively. The numbers of RSV patients in each age category were as follows: 0-5 months, 3373 (42.6%); 6-11 months, 1402 (17.7%); 12-23 months, 826 (10.4%); 2-4 years, 2095; 5-15 years, 213 (2.7%). The numbers of patients in each phase were as follows: Phase I, 1621(20.5%); and Phase II, 6288 (79.5%).

### Controls

The number of controls in pre-pandemic years 2016-2019, pandemic year 2020 and 2021 were 1264, 1316, 1200, 1185, 756, 779, respectively. During 2016 to 2019, the number of controls was stable in each year. However, in 2020 and 2021, the number of controls had an obvious decrease. Although the number of controls in 2021 was as low as that in 2020, the number of RSV patients had dramatically increased in 2021 (**Supplementary Figure 4**).

#### Change of positive rates of RSV infection

**Figure 3:**
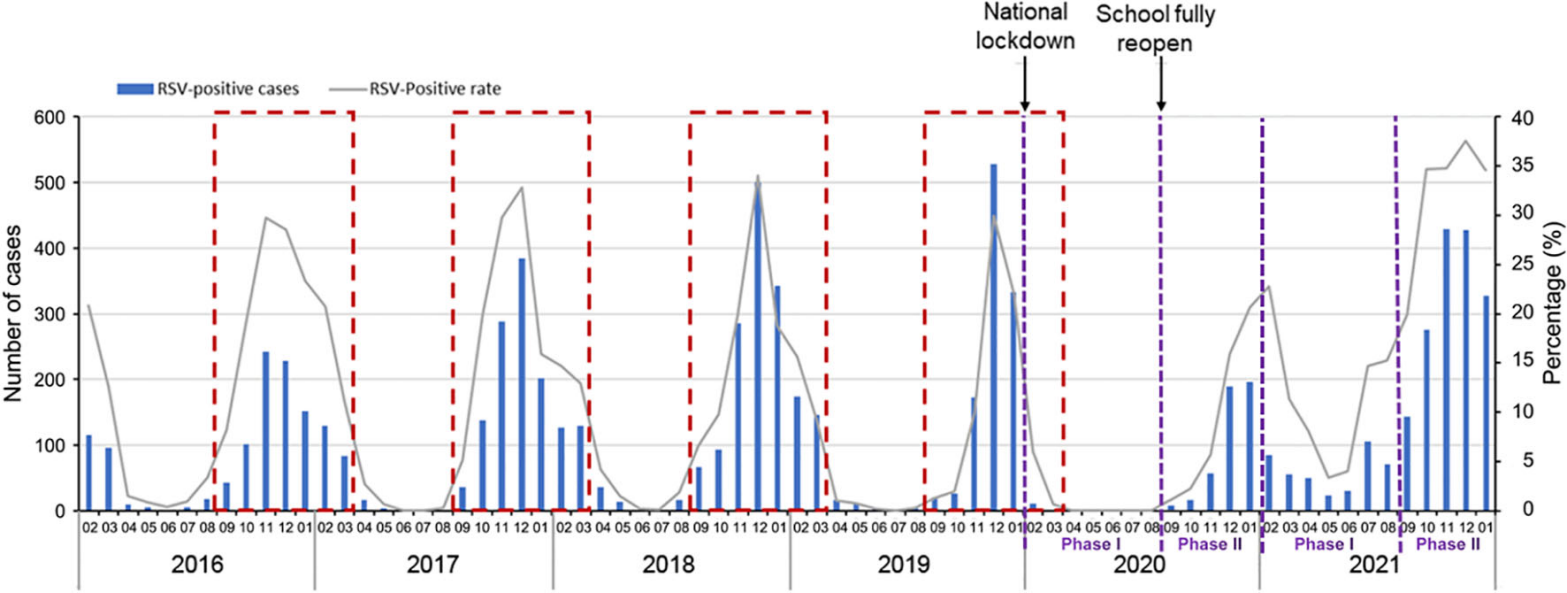
Annual RSV trends in Suzhou from the pre-pandemic years 2016−2019 to COVID-19 pandemic year 2020 and 2021. A study year is defined as February 1st of the current year to January 31st of the following year instead of the traditional calendar year. The dashed red boxes represent the typical RSV season (begins in September and runs through March of the following year).

During 2016 to 2019, the RSV cases at the study hospital followed the expected seasonal pattern (**Figure 3**). In 2020, after the start of the COVID-19 pandemic and the national lockdown in January, the number of RSV cases decreased sharply in February and March. There were no cases reported from April to August 2020. After the schools fully reopened in September 2020, the number of RSV cases gradually increased and peaked in December 2020 and January 2021 (**Figure 3**). The positive rate decreased from pre-pandemic years to 2020 by 85.1% (from 4.7% to 0.7%) in Phase I and 43.2% (from 18.3% to 10.4%) in Phase II. In 2021, the upsurge continued through May, the positive rate increased by 97.9% (from 4.7% to 9.3%) in Phase I, and 83.1% (from 18.3% to 33.5%) in Phase II compared to the same periods in pre-pandemic years (**Table 1**). Overall, the positive rate of RSV in 2020 decreased by 39.8%, whereas the positive rate in 2021 increased by 70.7% compared to the average level in the pre-pandemic years (**Table 1**).

**Table 1:** Clinical characteristics between the pandemic year 2021 and pre-pandemic years 2016−2019 for RSV.

	0-5 m		6-11 m		12-23 m		2-4y		5-15y	
	2016-2019	2021	2016-2019	2021	2016-2019	2021	2016-2019	2021	2016-2019	2021
Number of cases, n	2635	581	983	320	561	212	1079	827	79	125
Average year cases, n	658	581	246	320	140	212	270	827	20	125
Male sex, %	62.3	63.1	63.0	63.2	60.1	61.3	64.0	62.0	68.4	64.8
Median age, m	2 (1-3)	2 (1-3)	7.5 (6-10)	7 (6-9)	16 (14-18)	17 (15-20)	39 (29-49)	39 (29-45)	70 (56-88)	72 (60-84)
Comorbidities, %										
Cardiovascular diseases	8.5	7.4	2.1	3.8	1.4	2.8	1.0	1.1	0.2	1.6
Respiratory diseases	2	2.3	1.7	1.7	2.3	1.9	1.1	0.6	0	0
Neurologic and muscular diseases	0.1	0	1.2	1	1.4	1.2	1.2	1.4	6.3	4.8
Malignancy	0	0	0.8	1.2	1.2	0.9	1.1	1.0	1.2	3.2
Symptoms and signs on admission, %										
Fever (≥37.5°C)	18.9	15.3	39.3	57.1*	58.9	75.4*	77.4	87.1*	87.3	88.8
High fever (≥39°C)	2.3	4.2	15.2	24.5*	29.1	34.9*	58.0	63.8*	86.1	87.2
Cough	99.5	100	97.8	100	100	100	100	100	100	100
Wheeze	56.7	53.5	69.6	65.3	64.6	35.1*	43.9	27.6*	8.9	10.4
Rhinorrhea	58.8	62.0	60.8	63.4	59.7	62.7	54.0	57.1	54.4	57.6
Tachypnea	25.3	8.4*	17.3	14.3	15.2	11.8	10.2	6.9	3.8	2.4
Diarrhea or vomit	32.7	11.8*	45.0	20.4*	39.2	15.1*	30.9	16.0*	5.1	6.4
Feeding difficulty	23.9	9.5*	15.2	14.3	8.2	7.1	10.0	7.4	7.6	9.6
Coinfection with other viruses, %	4.1	10.1*	10.9	20.4*	17.8	20	7	15	22	20
Severe disease at admissions, %	21.3	7.1*	15.2	12.8	13.7	9.9	8.1	6.2	10.1	5.6
ICU admissions. %	10.9	4.6*	5.3	3.4	5.0	3.4	4.4	1.6	10.1	4.8
Oxygen support, %										
Any form	21.3	7.1*	15.2	12.8	13.7	9.9	8.1	6.2	10.1	5.6
High-flow nasal canula	7.1	4.3*	2.4	1.3	2.9	0.9	0.7	0.2	5.1	0
IMV	1.4	0	0.8	0	1.4	0.9	0.7	0.1	5.1	1.6
Death, %	0	0.2	0	0	0	0.9	0	0.1	1.2	0.8

*P<0.05 compared to the percentage in pre-pandemic years 2016−2019. The bold values means significant statistical differences before and after the epidemic.

**Figure 4:**
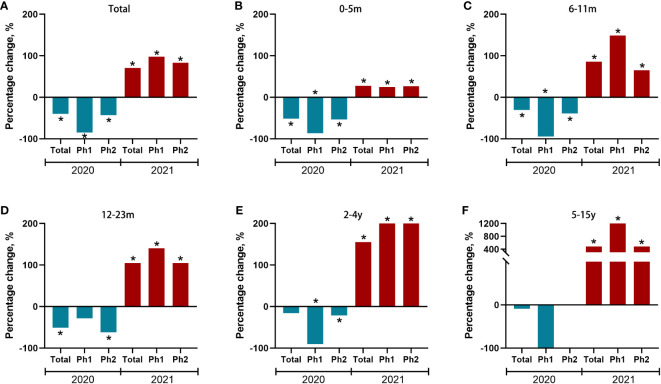
Percent change of test positive rate of RSV during the COVID-19 pandemic year 2020/2021 compared to the average incidences during pre-pandemic years 2016−2019 for each of two predefined periods and stratified by age group **(A-F)**. Red and blue bars indicate positive and negative percent changes, respectively. Statistically significant changes were marked with asterisks. Ph (Phase) 1: 1st February to Aug 31st, Ph (Phase) 2: Sep 1st to Dec 31th.

We further observed changes in positive rates of RSV among children in different age groups (**Figure 4** and **Supplementary Table 2**). In 2020, significant reductions of positive rates were observed in children aged 0-5 months, 6-11 months, and 12-23 months. In 2021, significant increases of positive rates were observed in all age groups. The largest increase of the annual cumulative positive rate was observed for children aged 5-15 years, an increase of 481.8% (from 1.1% to 6.4%), followed by 155.2% (8.7% to 22.2%) for 2-4 years, 104.8% (12.6%-25.8%) for 12-23 months, 85.7% (16.8%-31.2%) for 6-11 months, 27.6% (19.6%-25.0%) for 0-5 months.

#### Change of absolute number of RSV infections

We further stratified our analysis on the absolute number of RSV infections in different age groups. Interestingly, there was a significantly decrease in the number of RSV-related admissions in 2020 compared with the expected value in children aged 0 to 5 months (-76.8%; 95%CI, -82.3% to -66.4%, *P*<.01), 6-11m (-61.0%; 95%CI, -76.7% to -17.9%, *P*<.05) and 12-23m (-64.1%; 95%CI, -77.1% to -17.2%, *P*<.05), while there was an increase in the number of RSV-related admissions in 2021 in children aged 2-4y (149.1%; 95%CI, 67.7% to 378%, *P*<.01) and 5-15y (400.0%; 95%CI, 200.0% to 1460%, *P*<.05, **Table 2**).

**Table 2:** Comparison of RSV cases in 2020/2021 With the Average of 2016–2019 by Age.

	2016-2019	2020		2021	
	Average Annual Number	N	Difference From the Expected, % (95% CI)	N	Difference From the Expected, % (95% CI)
0-5m	658.8	157	-76.8 (-82.3 to -66.4) **	581	-14.1 (-34.4 to 24.4)
6-11m	245.8	99	-61.0 (-76.7 to -17.9) *	320	31.6 (-5.6 to 117.7)
12-23m	140.3	53	-64.1 (-77.1 to -17.2) *	212	43.4 (-8.2 to 231.2)
2-4y	269.8	189	-43.1 (-61.7 to 9.2)	827	149.1 (67.7 to 378.0) **
5-15y	19.8	9	-64.0 (-78.5 to 12.5)	125	400.0 (200.0 to 1460.0) *

**P<0.01.

*P<0.05.

#### Change of clinical characteristics of RSV infections between pre-pandemic years and pandemic year 2021

There were no significant differences between pre-pandemic years 2016-2019 and pandemic year 2021 in terms of sex and median age in each age groups (**Table 1**). Compared to the characteristics in the pre-pandemic years, significant increase of fever and high fever were observed in children aged 6-11 months (57.1% vs 39.3% for fever, and 24.5% vs 15.2% for high fever), 12-23 months (75.4% vs 58.9% for fever, and 34.9% vs 29.1% for high fever), and 2-4 years (87.1% vs 77.4% for fever, and 63.8% vs 58.0% for high fever) in 2021 (all *P*<0.05). Wheeze was significantly reduced in those aged 12-23 months (35.1% vs 64.6%, *P*<0.01), and 2-4 years (27.6% vs 43.9%, *P*<0.01). Tachypnea, and feeding difficulty were significantly reduced in those aged 0-5 months (8.4% vs 25.3% for tachypnea, and 9.5% vs 23.9%, both *P*<0.01). Diarrhea or vomit was significantly reduced in those aged 0-5 months (11.8% vs 32.7%, *P*<0.01), 6-11 months (20.4% vs 45.0%, *P*<0.01), 12-23 months (15.1% vs 39.2%, *P*<0.01), and 2-4 years (16.0% vs 30.9%, *P*<0.01). Coinfections with other viruses were significantly increased in those aged 0-5 months (10.1% vs 4.1%, *P*<0.01) and 6-11 months (20.4% vs 10.9%, *P*<0.01). Severe disease and ICU admissions were significantly reduced in those aged 0-5 months (21.3% vs 7.1% for severe disease, and 4.6% vs 10.9% for ICU admissions, both *P*<0.01).

#### Change of ICU admissions, Mortality and Comorbidities between pre-pandemic years and pandemic year 2021

The proportion of children who admitted to ICU decreased from 6.7% (n=376) in pre-pandemic years to 3.2% (n=66) in 2021 (*P*<0.01). We further compared the ICU proportions in different age groups, the downward trend was only significant in children aged 0-5m (10.9% vs 4.6%, **Table 1**). The proportions of high-flow oxygen therapy also decreased in 2021 (2.2% vs 1.2%, P<0.01). The downward trend of high-flow oxygen therapy was also only significant in children aged 0-5m (7.1% vs 4.3%, *P*<0.01, **Table 1**).

The number of RSV-associated death was relatively small. There was only one (0.02%) RSV-associated death (one with malignancy) in pre-pandemic years. However, there were 5 (0.2%) deaths (3 with malignancy, 1 with cardiovascular diseases, and 1 without comorbidities but die from necrotizing encephalitis) in 2021 (**Table 1**). The death rate of RSV infections in pre-pandemic years was 0.02%, while that in pandemic year 2021 was 0.2%. The detailed characteristics of RSV-associated death were described in **Supplementary Table 3**.

The clinical characteristics of the patients with RSV-associated ICU admissions between pre-pandemic years and pandemic year 2021 were also compared in **Table 3**. Patients in pandemic year 2021 was significantly older than those in pre-pandemic years (7 [2-24] vs 3 [2-6], *P*<0.001). Children aged >2 years accounted 31.8% of all the RSV-associated ICU admissions in 2021, which is significantly higher than that in the pre-pandemic years (10.4%). As comorbidities are risk factors for ICU admission. We also compare the proportions of the children with comorbidities. The proportion of children with cardiovascular diseases decreased from the average level of 17.0% in pre-pandemic years to 9.1% in 2021 (*P*<0.001), while the proportion of children with malignancy increased from the average level of 1.6% in pre-pandemic years to 12.1% in 2021 (*P*<0.001, **Table 3**).

**Table 3:** Clinical characteristics of patients need intensive care unit between the pandemic year 2021 and pre-pandemic years 2016−2019 for RSV.

	Pre-pandemic years (n=376)	Pandemic year 2021 (n=66)	P value
Male sex, %	248 (66.0)	42 (63.6)	0.72
Median age, m	3 (2-6)	7 (2-24)	**<0.001**
Age groups, %			
0-5m	272 (72.3)	27 (40.9)	**<0.001**
6-11m	47 (12.5)	12 (18.2)	0.21
12-23m	18 (4.8)	6 (9.1)	0.16
2-4y	32 (8.5)	12 (18.2)	**0.02**
5-15y	7 (1.9)	9 (13.6)	**<0.001**
Comorbidities, %	106 (28.2)	23 (34.9)	**<0.001**
Cardiovascular diseases	64 (17.0)	6 (9.1)	**<0.001**
Respiratory diseases	26 (6.9)	5 (7.6)	0.85
Neurologic and muscular diseases	18 (4.8)	3 (4.5)	0.93
Malignancy	6 (1.6)	8 (12.1)	**<0.001**
Metabolic diseases	6 (1.6)	2 (3.0)	0.34
Immunologic	3 (0.8)	2 (3.0)	0.16
Gastrointestinal	1 (0.3)	1 (1.5)	0.28
Renal	1 (0.3)	1 (1.5)	0.28
Complications during admission, %			
Pneumothorax	8 (2.1)	3 (4.5)	0.22
Pleural fluid	19 (5.1)	3 (4.5)	0.86
Meningitis	4 (1.1)	1 (1.5)	0.56
Seizure	4 (1.1)	2 (3.0)	0.22
Apnea	12 (3.2)	2 (3.0)	0.95
Oxygen support, %			
High-flow nasal canula	150 (39.9)	24 (36.4)	0.59
Mechanical Ventilation	49 (13)	11 (16)	0.44
Death, %	1 (0.3)	5 (7.6)	<0.001*

P<0.001 means the difference is statistically significant.

## Discussion

This study explored the change pattern of RSV infections during 2020 and 2021 in a low COVID-19 incidence area in Suzhou, southeast China. The activity of RSV was interrupted from February to August (Phase I) in 2020, when the most stringent epidemic prevention measures were in place. Several other studies have also found the RSV was inactive during this period (Partridge et al., 2021; Redlberger-Fritz et al., 2021; Yeoh et al., 2021; Zhang et al., 2021). When the community and school reopened in September 2020, RSV resurged gradually from September to December 2020, although the positive rate was lower than the average level in the same period during the pre-pandemic years. Previous studies have also reported a resurgence in RSV infections (Park et al., 2021; Weinberger Opek et al., 2021; Li et al., 2022; Jiang et al., 2023).

However, in 2021, the RSV positive rate increased markedly from the spring to winter, especially among children aged 2-4 years and 5-15 years. A recent study from Taiwan also found that the proportion of cases among children aged >2 years increased substantially (Lee et al., 2022). The mechanisms underlying the RSV resurgence in older children are unknown. The increasing number of RSV-naive children aged >2 years and decreased population immunity may have contributed to this resurgence (Lambert et al., 2014). Li et al. performed a multi-country observational study to identify the factors associated with RSV resurgence. They found that full reopening of schools and increased population susceptibility were associated with an increased risk of RSV resurgence (Li et al., 2022). School-age children are considered to be important transmitters of RSV (Graham, 2014; Munywoki et al., 2014; Jacoby et al., 2017). This phenomenon may also be due to the increasing number of children aged ≥2 years who were not infected by RSV when they were<2 years in 2020. This result highlights the risk of RSV outbreaks in children following transient RSV inactivity (Hall et al., 2013).

In 2021, children aged 6-11 months, 12-23 months and 2-4 years had significantly higher percentage of fever and high fever. While wheeze was significantly reduced in those aged 12-23 months, and 2-4 years. As RSV and IFV were highly active during the winter season in 2021. The large number of children aged ≥2 years with high fever, but no wheezing actually made it difficult for paediatricians to differentiate RSV and IFV during the winter season in 2021.

Our study provides evidence that the resurgence of RSV during the COVID-19 epidemic is associated with less severe RSV infections. We found an overall reduction in severe diseases and ICU admissions in 2021. The downward trend was only significant in children aged 0-5m after we further compared the proportions in different age groups. The most recent studies in Europe and Australia found that the resurgence of RSV was not associated with an increased risk of severe disease (Fourgeaud et al., 2021; Foley et al., 2022; Saravanos et al., 2022), whereas a study from USA found an increased percentage of ICU admission (Agha and Avner, 2021). However, all these studies had not made further comparisons in different age groups. More research is warranted to explore the impact of the resurgence in the incidence of RSV infection on disease severity in different regions. Although severe diseases and ICU admissions were reduced in 2021, we could not ignore the fact that RSV-associated death increased in 2021 (0.2% vs 0.02%), especially for those with comorbidities.

As comorbidities are risk factors for ICU admission. We also compare the proportions of the comorbidities in RSV-associated ICU admissions. The proportion of children with cardiovascular diseases decreased from the average level of 17.0% in pre-pandemic years to 9.1% in 2021 (*P*<0.001), while the proportion of children with malignancy increased from the average level of 1.6% in pre-pandemic years to 12.1% in 2021 (*P*<0.001). Most of children with cardiovascular diseases and severe RSV infections are less 6 months. The decrease of comorbidities of cardiovascular diseases may explain the downward trend of ICU admissions in children aged 0-5m. Children with malignancies are usually older than 2 years. As the increase trend in RSV infections were more apparent in those aged 2-4 years and 5-15 years in our study, those with malignancies would have an increase odds of RSV infections. Children with malignancies usually had a high death rate when they get infected. This could explain the increased death rate in this RSV resurge. Thus, paediatricians should pay more attentions to the increase of children with malignancies and severe RSV infections.

Strengths of this study include the use of a large number of cases stratified by five age groups, different clinical characteristics, and severity to compare the RSV epidemiology in the second year after the onset of the COVID-19 pandemic with that in the four pre-pandemic years. Besides, our PCR assay had great performance with 86.5%-100% positive prediction value and 97.8%-99.85% negative prediction value (Li et al., 2019; Zhang et al., 2020). This study has some limitations. First, we did not investigate climatic factors such as temperature and humidity, which might have played a role in the RSV resurgence (Baker et al., 2019). Second, we did not test the RSV genotype, thus any change of RSV genotype or virulence in this unusual RSV resurgence was not determined. Third, RSV was only tested in hospitalized patients, the data in our study can only represent the status of inpatients, but cannot understand the overall RSV-associated activity in our area. Fourth, we tested for only six common viruses. This may have underestimated the true coinfection burden of RSV in our region.

**Figure 5:**
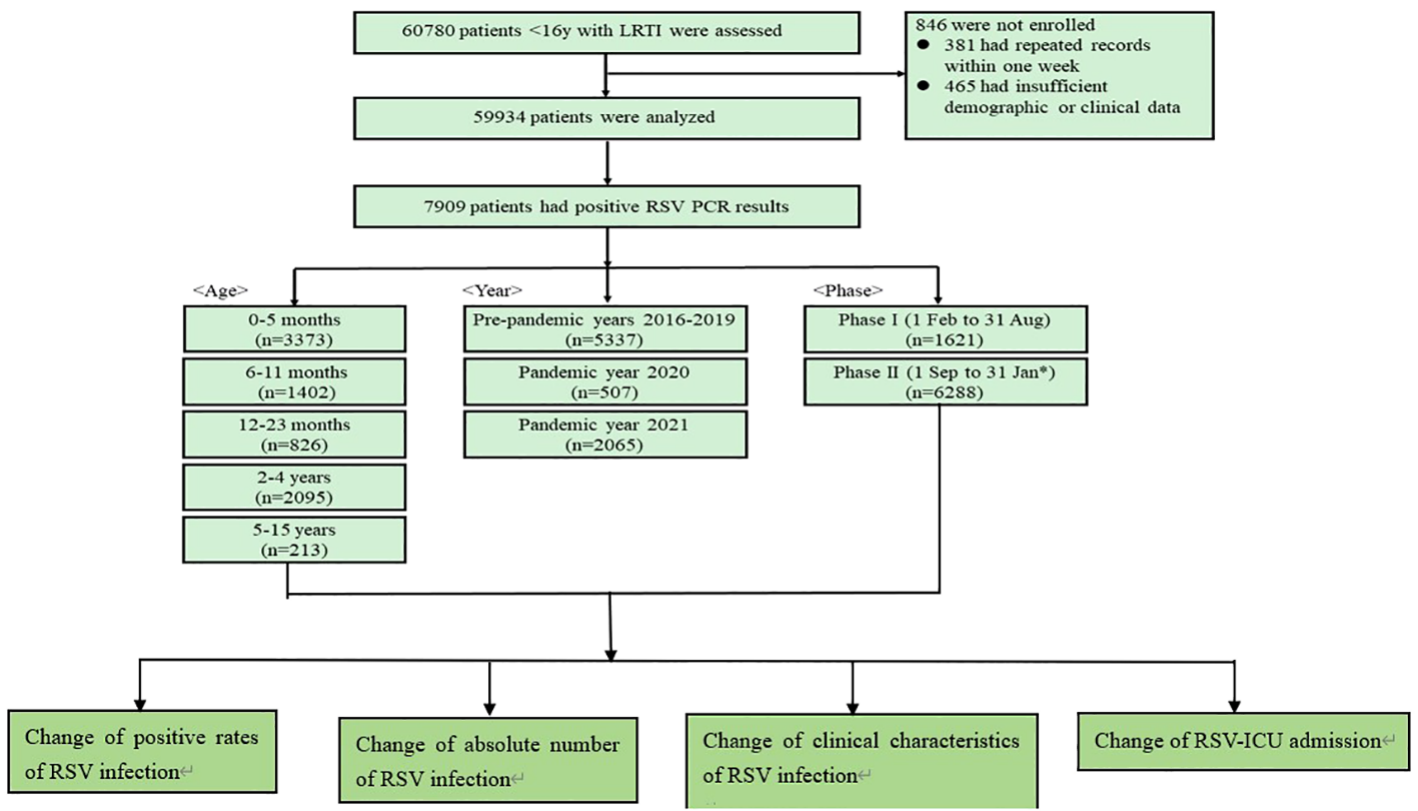
A summary of the results of this study.

In summary (**Figure 5**), our findings raise concerns regarding RSV control in Suzhou, southeast China. The COVID-19 public health measures were associated with a decreased number of RSV infections during the first year of the pandemic. However, during the second year of the pandemic, the gradual reduction in COVID-19-related public health measures caused a significant increase in the number of RSV infections. School reopening and a build-up in population susceptibility may account for the resurgence of RSV. The resurgence of RSV was more apparent in children aged >2 years and was associated with a higher percentage fever, but less percentage of wheeze. Although severe disease and ICU admissions were significantly reduced in this resurgence, we could not ignore the increase of RSV-associated death in 2021.

## Data availability statement

The raw data supporting the conclusions of this article will be made available by the authors, without undue reservation.

## Ethics statement

The studies involving humans were approved by Medical Ethics Committee of the Children’s Hospital of Soochow University (2013002). The studies were conducted in accordance with the local legislation and institutional requirements. The human samples used in this study were acquired from primarily isolated as part of your previous study for which ethical approval was obtained. Written informed consent for participation was not required from the participants or the participants’ legal guardians/next of kin in accordance with the national legislation and institutional requirements.

## Author contributions

WJ and SC wrote the manuscript together and contributed equally. ML, ZZ and ZW completed the data collection and analysis. XS and SH was responsible for the detection of specimens. YW and CH designed the study and reviewed the manuscript. All authors contributed to the article and approved the submitted version.
