# Supplementary material for: Are we ready to face the next wave of RSV surge after the COVID-19 Omicron pandemic in China?
Source: Front Cell Infect Microbiol. 2023 Dec 12;13:1216536. doi: 10.3389/fcimb.2023.1216536 (PMC10751930; doi:10.3389/fcimb.2023.1216536)
Supplement: Supplementary file 1 [file DataSheet_1.docx]

**Supplementary Materials**

**Potential Threats to Older Children from Respiratory Syncytial Virus Infections After the First Wave of COVID-19 Pandemic in Suzhou, China**

**Supplementary Methods**

***Etiological surveillance of low respiratory tract infections (LRTI) in children, Suzhou, Southeast China.***

This is an ongoing, prospective, active surveillance, initiated in 2013. The current report deals with data from Feb 2016 through Jan 2022. All children <16 years with LRTI hospitalized in two branches (General Hospital, Jingde Road Branch) of Children’s Hospital of Soochow University were included in the analysis. LRTI was defined as at least one of the following manifestations: cough, nasal flaring, indrawing of the lower chest wall, subcostal retractions, stridor, rales, rhonchi, wheezing, crackles or crepitations, or observed apnea. Children’s Hospital of Soochow University is a tertiary children’s hospital in Suzhou, Southeast China. The hospital has 1500 beds reserved for children.

***Clinical specimen***

Nasopharyngeal aspirates were obtained from all the inpatients within 24 hours after admission. This involved passing a suction catheter through the nose with the intent of passing it into the lower part of the pharynx. The depth of penetration for the nasopharyngeal aspirate catheter was set at 7-9 cm. A total of 2 ml nasopharyngeal aspirates was obtained and sent for analysis within 30min. It is centrifuged at 500×g for 10 minutes and resuspended in 2 ml saline supernatant for multiple respiratory pathogens detecting. Total nucleic acid (DNA and RNA) was extracted from the collected specimen. Nucleic acid extracts were then tested for six viruses including RSV, human rhinovirus (HRV), influenza virus (IFV), parainfluenza virus (PIV), adenovirus (ADV), human metapneumovirus (HMPV) using the multiple respiratory pathogen panel assays (Health Gene Technologies, Ningbo, China). The primer/probes used and amplification condition were provided upon request. All steps of the nucleic acid extraction and PCR/RT-PCR test were conducted in parallel with positive and negative controls.

**Supplementary Table 1.** Demographic characteristics compared between acute low respiratory tract infections patients in pre-pandemic years (2016‒2019) and the two COVID-19 pandemic years (2020-2021).

|  | Pre-pandemic years (2016‒2019) | COVID-19 year  2020 | COVID-19 year  2021 |
| --- | --- | --- | --- |
| Total number | 43268 | 6833 | 9833 |
| Sex |  |  |  |
| Male | 25138 (58.1) | 4031 (59.0) | 5634 (57.3) |
| Female | 18130 (41.9) | 2802 (41.0) | 4199 (42.7) |
| Age group |  |  |  |
| 0-5 months | 13463 (31.1) | 1657 (24.2) | 2321 (23.6) |
| 6-11 months | 5868 (13.6) | 846 (12.4) | 1025 (10.4) |
| 12-23 months | 4468 (10.3) | 854 (12.5) | 820 (8.3) |
| 2-4 years | 12406 (28.7) | 2572 (37.6) | 3720 (37.8) |
| 5-15 years | 7063 (16.3) | 904 (13.2) | 1947 (19.8) |
| ICU admissions | 2376 (5.5) | 389 (5.7) | 520 (5.3) |

Data are n (%) unless otherwise indicated.

**Supplementary Table 2.** Comparison of a positive rate (%) of RSV between pre-pandemic years (2016‒2019) and the COVID-19 pandemic year 2020/2021 in Suzhou, Southeast China.

|  |  | Total |  |  |  | Phase Ⅰ† |  |  |  | Phase Ⅱ† |  |
| --- | --- | --- | --- | --- | --- | --- | --- | --- | --- | --- | --- |
| 2020 |  |  |  |  |  |  |  |  |  |  |  |
|  | 2016-2019 | 2020 | Relative change |  | 2016-2019 | 2020 | Relative change |  | 2016-2019 | 2020 | Relative change |
| Total | 12.3 | 7.4 | -39.8%* |  | 4.7 | 0.7 | -85.1%* |  | 18.3 | 10.4 | -43.2%* |
| 0-5M | 19.6 | 9.5 | -51.5%* |  | 8.9 | 1.2 | -86.5%* |  | 30.1 | 14.0 | -53.5% * |
| 6-11M | 16.8 | 11.7 | -30.4% * |  | 7.2 | 0.4 | -94.4% * |  | 27.7 | 16.9 | -39.0% * |
| 12-23M | 12.6 | 6.2 | -50.8%* |  | 3.5 | 2.5 | -28.6% |  | 19.2 | 7.3 | -62.0% * |
| 2-4y | 8.7 | 7.3 | -16.1% |  | 3.2 | 0.3 | -90.6% * |  | 12.2 | 9.6 | -21.3%* |
| 5-15y | 1.1 | 1.0 | -9.1% |  | 0.2 | 0.0 | -100.0% |  | 1.8 | 1.8 | 0.0% |
| 2021 |  |  |  |  |  |  |  |  |  |  |  |
|  | 2016-2019 | 2021 | Relative change |  | 2016-2019 | 2021 | Relative change |  | 2016-2019 | 2021 | Relative change |
| Total | 12.3 | 21.0 | 70.7%* |  | 4.7 | 9.3 | 97.9%* |  | 18.3 | 33.5 | 83.1%* |
| 0-5M | 19.6 | 25.0 | 27.6%* |  | 8.9 | 11.1 | 24.7%* |  | 30.1 | 38.1 | 26.6% * |
| 6-11M | 16.8 | 31.2 | 85.7%* |  | 7.2 | 17.9 | 148.6% * |  | 27.7 | 45.7 | 65.0% * |
| 12-23M | 12.6 | 25.8 | 104.8%* |  | 3.5 | 8.4 | 140.0% * |  | 19.2 | 39.3 | 104.7%* |
| 2-4y | 8.7 | 22.2 | 155.2%* |  | 3.2 | 9.6 | 200.0% * |  | 12.2 | 37.8 | 209.8%* |
| 5-15y | 1.1 | 6.4 | 481.8%* |  | 0.2 | 2.6 | 1200.0%* |  | 1.8 | 10.5 | 483.3% * |

*Statistically significant changes were found (p-value<0.05 based on chi-square test).

†Phase Ⅰ: 1st Feb to 31st Aug; Phase Ⅱ: 1st Sep to 31st Jan of the following year.

**Supplementary Table 3. The detailed characteristics of RSV-associated death from 2016-2021.**

| Year | Sex | Age | Comorbidities | Death Reason |
| --- | --- | --- | --- | --- |
| 2016 | - | - | - | - |
| 2017 | - | - | - | - |
| 2018 | - | - | - | - |
| 2019 | Male | 10y | Malignancy | Malignancy and sepsis |
| 2020 | - | - | - | - |
| 2021 | Female | 5m | Congenital heart disease | Respiratory and heart failure |
|  | Male | 12m | Malignancy | Malignancy and sepsis |
|  | Male | 15m | Malignancy | Malignancy and sepsis |
|  | Female | 11y | Malignancy | Malignancy and sepsis |
|  | Male | 3y | N/A | Necrotizing encephalitis |

**Supplementary Figure 1. Monthly reported COVID-19 cases in Suzhou during the COVID-19 epidemic years 2020-2021.** L1,2,3 indicated level 1,2,3 public health emergency response.


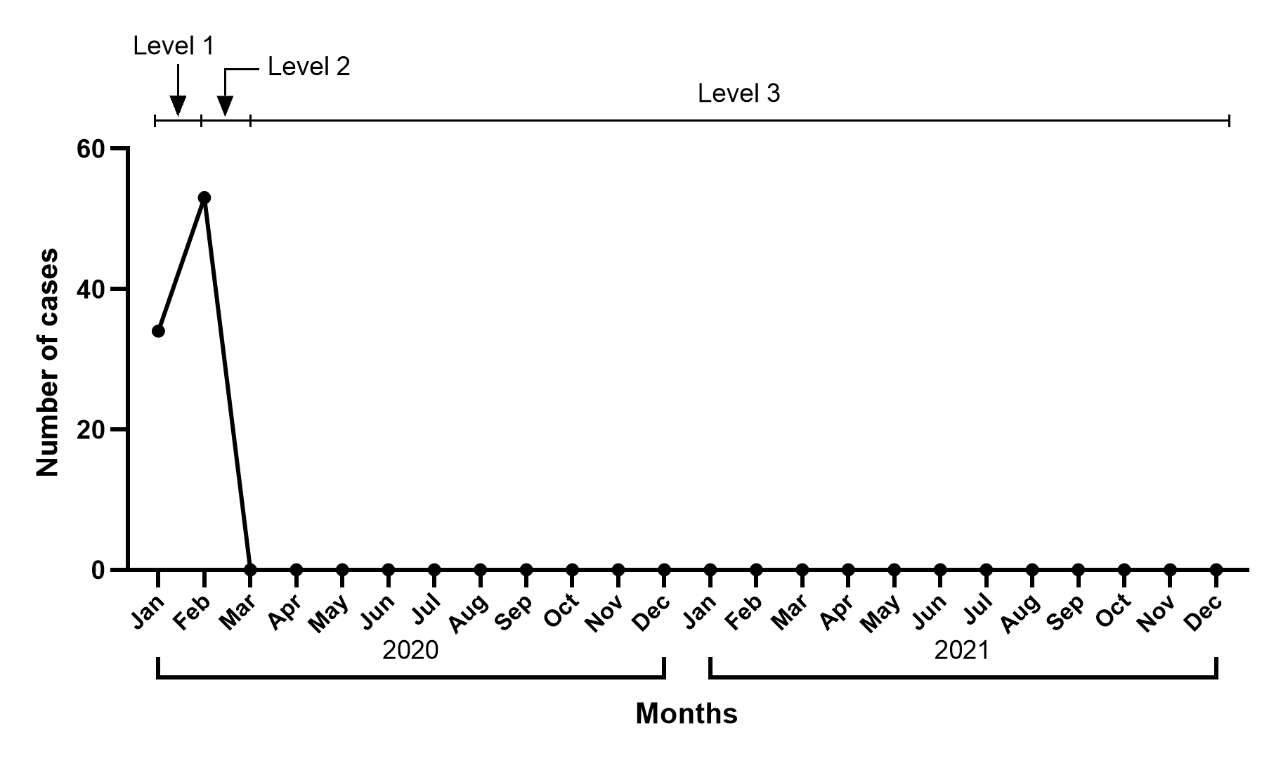


**Supplementary Figure 2. Phase I and II in the COVID-19 epidemic year 2020/2021.** Two periods in COVID-19 epidemic year 2020 were defined according to the timeline of major intervention events for containing the COVID-19 epidemic and seasonal pattern of RSV: 1st February to Aug 31st (Phase I), Sep 1st to Dec 31th (Phase II). The same periods were also defined based on corresponding intervals for 2021. The black dotted line indicated the average monthly percentages during the pre-pandemic years 2016-2019. Level 1,2,3 indicated level 1,2,3 public health emergency response.


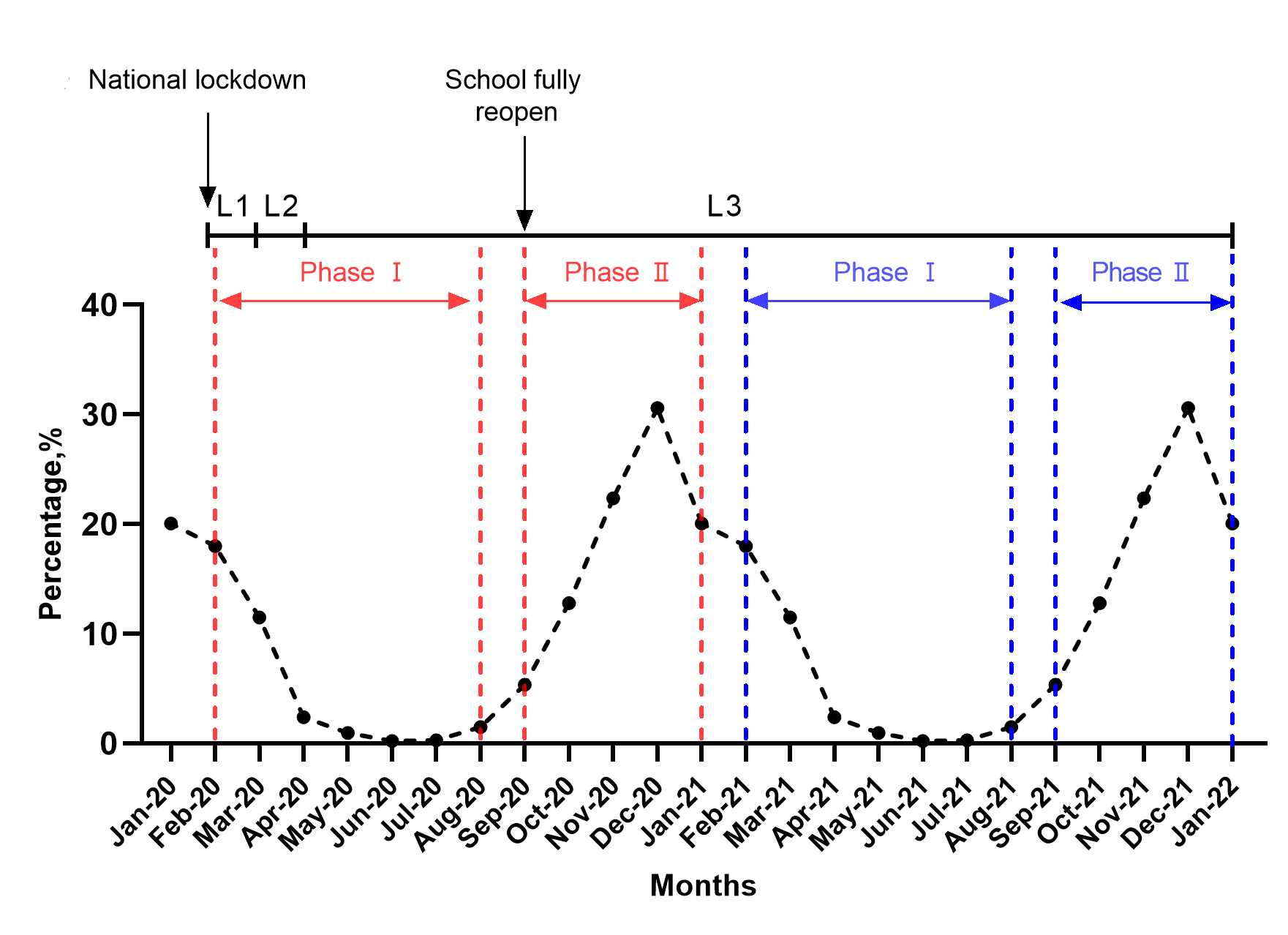


**Supplementary Figure 3. Annual numbers of patients enrolled in our study.** A study year is defined as February 1^st^ of the current year to January 31st of the following year instead of the traditional calendar year.


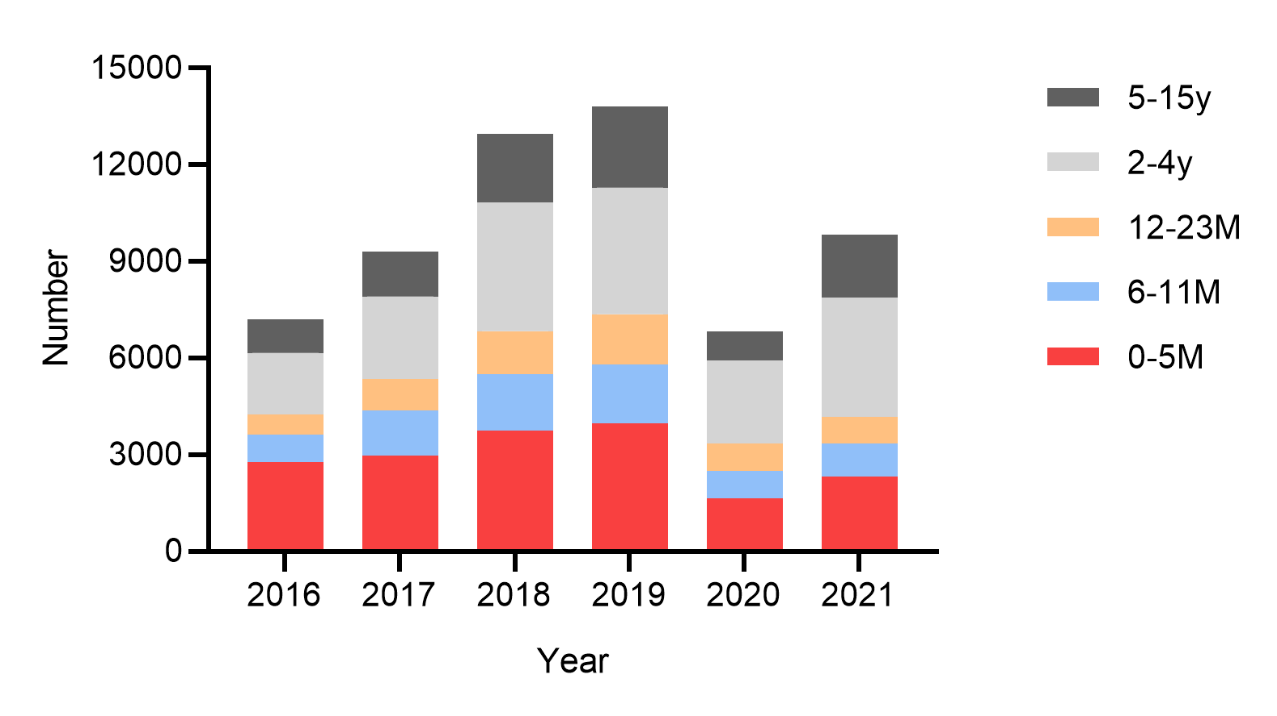


**Supplementary Figure 4. Annual numbers of controls and RSV cases in our study.** A study year is defined as February 1^st^ of the current year to January 31st of the following year instead of the traditional calendar year.

**
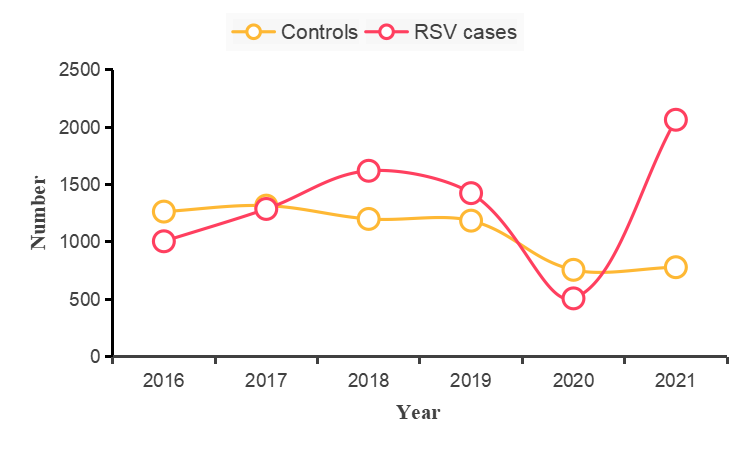
**
